# Kidney injury molecule type-1, interleukin-18, and insulin-like growth factor binding protein 7 levels in urine to predict acute kidney injury in pediatric sepsis

**DOI:** 10.3389/fped.2022.1024713

**Published:** 2022-12-05

**Authors:** Idham Jaya Ganda, Yusriwanti Kasri, Maya Susanti, Fitrayani Hamzah, Syarifuddin Rauf, Husein Albar, Jusli Aras, Bahrul Fikri, Sitti Aizah Lawang, Dasril Daud, Amiruddin Laompo, Muhammad Nasrum Massi

**Affiliations:** ^1^ Emergency and Pediatric Intensive Care Division, Department of Pediatrics, Faculty of Medicine Hasanuddin University, Makassar, Indonesia; ^2^ Department of Child Health, DR Wahidin Sudirohusodo Hospital, Makassar, Indonesia; ^3^ Department of Pediatrics, Faculty of Medicine Hasanuddin University, Makassar, Indonesia; ^4^ Nephrology Division, Department of Pediatrics, Faculty of Medicine Hasanuddin University, Makassar, Indonesia; ^5^ Allergy and Immunology Division, Department of Pediatrics, Faculty of Medicine Hasanuddin University, Makassar, Indonesia; ^6^ Hematology-Oncology Division, Department of Pediatrics, Faculty of Medicine Hasanuddin University, Makassar, Indonesia; ^7^ Respirology Division, Department of Pediatrics, Faculty of Medicine Hasanuddin University, Makassar, Indonesia; ^8^ Department of Microbiology, Faculty of Medicine Hasanuddin University, Makassar, Indonesia

**Keywords:** AKI, sepsis, urinary biomarker, KIM-1, IL-18, IGFBP-7, PICU

## Abstract

**Background:**

This study aimed to observe the role of urinary kidney injury molecule (KIM-1), interleukin (IL-18), and insulin-like growth factor-binding protein 7 (IGFBP-7) levels in predicting acute kidney injury (AKI) in children with sepsis.

**Material and Methods:**

This prospective cohort observational study was conducted at Dr. RSUP. Wahidin Sudirohusodo, Makassar, South Sulawesi, from January to December 2021. Inclusion criteria were septic patients treated in the pediatric intensive care unit (PICU) aged 1 month to 18 years with normal serum creatinine or normal urine output (>5 ml/kg/body weight (BW)/h in 6–12 h). Patients with a history of kidney disease, prior urinary tract infection, or history of using nephrotoxic drugs were excluded.

**Results:**

There was a significant difference in urinary KIM-1, IL-18, and IGFBP-7 levels between septic patients with and without AKI. The cut-off point for urinary KIM-1 level in sepsis with and without AKI was 1.666 ng/ml, with sensitivity of 82.5%, specificity of 82.2%, and a relative risk (RR) [95% confidence interval (CI)] of 6.866 (95% CI, 3.329–14.165). The cut-off point for urinary IL-18 levels was 3.868 ng/ml, with sensitivity of 92.50%, specificity of 91.78%, and RR of 20.078 (95%CI, 6.593–61.142). The cut-off point for urinary IGFBP-7 levels was ≥0.906 ng/ml with a sensitivity of 75.00%, specificity of 75.34%, and RR of 4.063 (95% CI, 2.206–7.483).

**Conclusion:**

Urinary KIM-1, IL-8, and IGFBP-7 levels could be used to predict AKI in septic patients. Urinary IL-8 has a higher sensitivity and specificity as a predictor of AKI in patients with sepsis.

## Introduction

**Figure 1:**
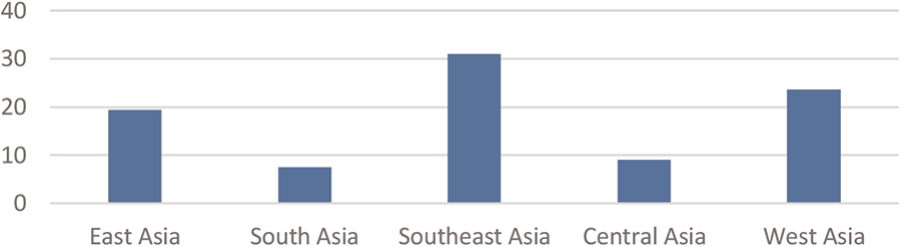
Incidence of AKI in Asia. AKI, acute kidney injury.

Acute kidney injury (AKI) is still a cause of morbidity and mortality in children (1). The incidence of AKI due to sepsis is around 45%–75% (2, 3). The risk of death or disability is higher in children with AKI and severe sepsis (4). According to research conducted in Asia, the prevalence of AKI is high (Figure 1). The mortality of AKI is highest in the East Asia (36.9%), followed by South Asia (13.8%), and West Asia (23.6%) (5). There are no national data of AKI in children in Indonesia (6). A previous study in Makassar during 2017–2018 showed that the AKI prevalence due to sepsis in children was 40% (7).

The diagnosis of AKI in children is confirmed by three parameters: an increase in serum creatinine, a decrease in the glomerular filtration rate (GFR), or a decrease in urine production within 24 h (1). A serum creatinine test is a routine examination that is frequently used to assess kidney function easily, rapidly, and affordable (8). However, the serum creatinine test has limitations in that it is not sensitive to small changes in the GFR, it does not change until 50% of kidney function is damaged, and it rises up to 72 h after injury. Serum creatinine concentration is influenced by age, sex, muscle mass, and volume status (1).

Over the past two decades, many researchers have been looking for new biomarkers that are more sensitive, specific, affordable, and noninvasive (1, 9), including biomarkers that can predict AKI (10, 11). Several biomarkers for early detection of AKI include urinary tubular enzymes (glutathione-S-transferase, gamma-glutamyl transferase, N-acetyl-*β*-D-glucosaminidase), low molecular weight urine protein (urinary cystatin C), markers of urinary inflammation (interleukin (IL), IL-1, IL-6, IL-8, IL-18, matrix metalloproteinase-2), proximal tubular response (neutrophil gelatinase-associated lipocalin (NGAL), kidney injury molecule (KIM-1), cystatin C, liver type fatty acid binding protein and protein in 5 arrest cell cycle [insulin-like growth factor-binding protein 7 (IGFBP-7)], and tissue inhibitor of metalloproteinases-2 (11–13).

This study chose KIM-1, IL-18, and IGFBP-7 over other urinary biomarkers for the following reasons. IL-18 levels are physiologically deficient but can be increased several fold in patients with renal injury (14). Increased IL-18 levels in urine are caused by tubular injury in septic patients (15). IGFBP7 can induce cell cycle insults in G1 phase by directly increasing P21 and P53 expression that occurs in renal tubular epithelial cells during renal injury caused by ischemia or sepsis. This IGFBP7 has a sensitivity of about 88%–96% for detecting ARF in the early stages (16). KIM-1, according to a study by Ta, showed that KIM-1 increased significantly in the first 6 h, peaked at 24 h, and continued to increase in significance up to 48 h after ICU admission (17).

Sepsis is a life-threatening organ dysfunction caused by dysregulation of the immune system against infection. Evidence of infection was obtained from physical examination, radiological examination, laboratory examination, and blood culture (18). Because of the high incidence of AKI due to sepsis, the difficulty in making a diagnosis, and the poor impact that arises from late diagnosis, it is essential to conduct a study to determine the role of urinary KIM-1, IL-18, and IGFBP-7 biomarkers in predicting the AKI occurrence in children with sepsis. So, it is expected to provide benefits in terms of faster treatment and management, reduced costs and length of treatment, reduced mortality, and contribute to increased expertise and clinical experience in the diagnosis and management of AKI in children**.**

There has never been a multicenter study in eastern Indonesia on KIM-1, IL-18, and IGFBP-7 in urine as biomarkers to predict the occurrence of AKI in children treated with sepsis in the pediatric intensive care unit (PICU) in Makassar, South Sulawesi. The purpose of this study is to determine the role of urinary KIM-1, IL-18, and IGFBP-7 levels in predicting the occurrence of AKI in children with sepsis**.**

## Materials and methods

This prospective cohort observational study was conducted at Dr. RSUP. Wahidin Sudirohusodo, Makassar, South Sulawesi, from January to December 2021. Research permit was obtained from the Wahidin Sudirohusodo Hospital and Ethical approval from the Commission for Biomedical Research Ethics in Humans, Faculty of Medicine, Hasanuddin University.

The inclusion criteria were septic patients treated in the PICU, aged 1 month to 18 years, with normal serum creatinine or normal urine output (0.5**–**1.5** **ml/kg/ body weight (BW) h) (19). Patients with a history of kidney disease, previous urinary tract infection, and a history of using nephrotoxic drugs, such as aminoglycosides, amphotericin B, radiologic contrast, and drugs impairing renal hemodynamic like angiotensin converting enzyme inhibitors and angiotensin II receptor blockers, in 1–5 days before admission were excluded.

Estimating sample size using the sample size formula for proportion comparison, if relative risk (RR) of 1.75 is considered significant, the proportion in the sepsis group with AKI is 20%, with a significance value of 0.05 and a power of 80%. The sample size is calculated as [1.96√2(0.3** **×** **00.7)** **+** **0.842√0.4** **×** **0.6** **+** **0.2** **×** **0.8]2/ (0.4–0.2)2** **=** **80.68, so the minimum sample size is 80. The sample was collected using the consecutive sampling method. The subjects were observed to obtain the outcome, with and without AKI, which was determined using the Kidney Disease Improving Global Outcomes criteria (KDIGO)**.**

Serum creatinine levels were assessed in the laboratory of Wahidin Sudirohusodo Hospital on the first day of PICU treatment and then every 2 days subsequently. The middle stream urine sample was collected aseptically in the morning using a falcon tube of 15–20 ml to measure the levels of KIM-1, IL-18, and IGFBP-7. The urine sample was centrifuged to remove particulate matter and collect the supernatant, and then was stored at −20°C. Before analyzing urine KIM-1, IL-18, and IGFBP-7 levels, samples were stored at room temperature (18–25°C) without additional warmers and mixed well by turning gently before pipetting. 100 µl of standard and sample were added to the tap hole, covered with a plate sealer, and incubated at room temperature for 2.5 h with gentle vibration. After that, liquid was aspirated from each container and washed four times. In each well, 100 µl of biotinylated detection antibody was added, gently shaken, and incubated at room temperature for 1 h. 100 µl of horseradish peroxidase (HRP)–streptavidin solution was added and incubated at room temperature for 45 min. 100 µl of tetramethylbenzidine (TMB) substrate solution was added and incubated for 30 min at room temperature with gentle vibration. Finally, a 50 µl stop solution was poured into each well, and the optical density (OD value) of each well was immediately measured with a 450 nm microplate reader. The urine KIM-1, IL-18, and IGFBP-7 levels were measured and reported in ng/ml (20). Urine KIM-1 levels were measured using the Human KIM-1 ELISA Kit, urine IL-18 levels with the Human Interleukin 18 Enzyme-Linked Immunosorbent Assay Kit, and urinary IGFBP-7 levels using the Human Lip IGFBP-7 ELISA Kit.

KDIGO 2012 defines acute kidney injury as any of the following: increase in serum creatinine by 0.3 mg/dl or more within 48 h; increase in serum creatinine to 1.5 times baseline or more within the last 7 days; or urine output <0.5 ml/kg/h for 6–12 h (15). Normal serum creatinine levels in children aged 1 month to 1 year are 0.2**–**0.4 mg/dl, in children aged >1 year to <12 years are 0.3**–**0.7 mg/dl, and in children aged 12 to <18 years are 0.5**–**1.2 mg/dl (16).

Data were entered into Microsoft Excel 2021 and analyzed using SPSS version 21. Univariate analysis for data description was in the form of frequency distribution, mean, standard deviation, median, and range. Bivariate analysis used Student’s *t*-test, Mann–Whitney *U* test, chi-square test, and Fisher exact test. The Kolmogorov–Smirnov test was used to determine data normality, and the Levene test was used to determine variance similarity. The cut-off point for urine KIM-1, IL-18, and IGFBP7 levels was determined clinically and statistically as the boundary between sepsis with and without AKI, making the receiver operating characteristic (ROC) curve to determine area under the curve (AUC) and determine the sensitivity, specificity, positive predictive value (PPV), negative predictive value (NPV), positive likelihood ratio (LR+), negative likelihood ratio (LR−), accuracy, and RR with 95% confidence interval (95% CI). The hypothesis test results are not significant if *p*** **>** **0.05 and significant if *p*** **<** **0.05.

## Results

**Figure 2:**
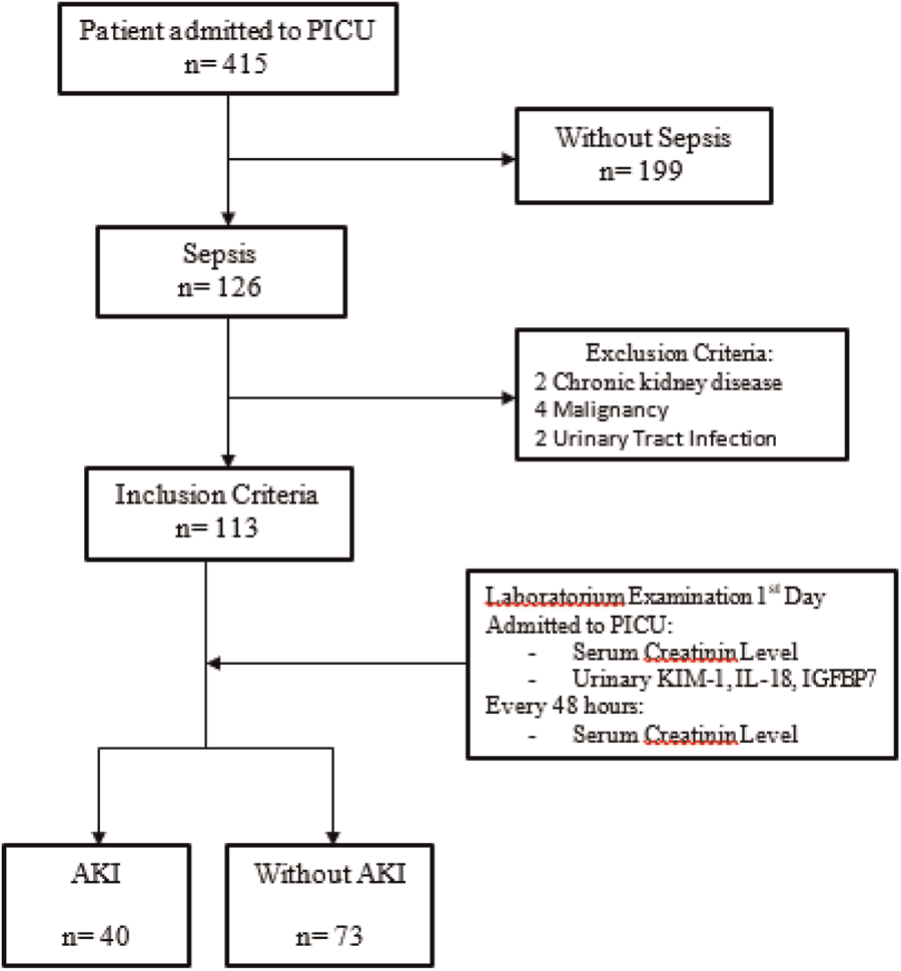
Study flowchart.

A total of 415 patients were treated in the PICU from January to December 2021, with 126 septic patients and 199 non-septic patients. Only 113 of the 126 septic patients met the inclusion criteria, with 40 (35.4%) having AKI and 73 (64.6%) not having AKI. The remaining 13 patients were not included because they had chronic kidney disease, cancer, or urinary tract infections (Figure 2).

Septic patients in children are more dominant in male (64.60%) at the age of <5 years (57.52%). Primary diseases causing sepsis consisted of pneumonia in 36 patients (31.9%), tuberculosis cases in 5 patients (4.4%), cases of gastrointestinal surgery in 19 patients (16.8%), neurosurgery cases in 11 patients (9.7%), meningitis in 11 patients (9.7%), surgical cases in orthopedics in 2 patients (1.8%), status epilepticus in 4 patients (3.5%), gastroenterology disease in 3 patients (2.7%), epilepsy cases in 3 patients (2.7%), congenital heart disease in 2 patients (1.8%), and burns in 1 patient (0.9%) (Table 1).

**Table 1:** Characteristics of the septic patients on the first day of treatment in the PICU.

Sample characteristics	Sepsis with AKI *n* = 40 (35.4%)	Sepsis without AKI *n* = 73 (64.6%)	*P*
Age <5 years	29 (44.6%)	36 (55.4%)	0.071^a^
Male	24 (32.9%)	49 (67.1%)	0.538^a^
Malnutrition	15 (30.6%)	34(69.4%)	0.352^a^
Anemia	19 (34.5%)	36 (65.6%)	0.854^a^
Leukopenia/leucocytosis	24 (30%)	56 (70%)	0.062^a^
CRP ≥10** **mg/L	26(35.1%)	48 (64.9%)	0.936^a^
Procalcitonin ≥1** **mg/ml	28 (41.2%)	40 (58.8%)	0.114^a^
Positive blood culture	7 (33.3%)	15 (66.7%)	0.826^a^
PELOD-2 score ≥10	35 (36.4%)	61 (63.6%)	0.850^a^
Mechanical ventilation	17 (41.5%)	24 (58.5%)	0.309^a^
Primary disease, *n* (%)			
Pneumonia	36 (31.9)		
Tuberculosis	5 (4.4)		
Gastrointestinal surgery	19 (16.8)		
Neurosurgery	11 (9.7)		
Meningitis	11 (9.7)		
Orthopedic surgery	2 (1.8)		
Infection disease	16 (14.2)		
Status epilepticus	4 (3.5)		
Gastrointestinal disease	3 (2.7)		
Epilepsy	3 (2.7)		
Congenital heart disease	2 (1.8)		
Burns	1 (0.9)		

AKI, acute kidney injury.

Malnutrition: with CDC chart >5 years old and with WHO chart <5 years old, anemia (≤11 g/dl), leukopenia (<4.0 × 10^9^/L), leukocytosis (>11.0 × 10^9/L), and CRP (Normally < 10 mg/dl). Gastrointestinal surgery: Appendicitis, Hirschsprung's disease, intussusception, and abdominal tumor. Neurosurgery: hydrocephalus, traumatic brain injury, and fracture cranium. Orthopedic surgery: scoliosis and neglected at hip joint. Infection disease: typhoid fever and dengue shock syndrome. Gastrointestinal disease: acute diarrhea with dehydration and liver abscess. Congenital heart disease: Cyanotic spell due to tetralogy of fallot.

^a^ Chi-square.

The mean (SD) levels of KIM-I, IL-18, and IGFBP-7 in septic patients with AKI were 1.92 (0.30), 4.75 (0.71), and 1.06 (0.59), respectively. Meanwhile, the mean (SD) level of KIM-I, IL-18, and IGFBP-7 in septic patients without AKI were lower than those with AKI, with 0.96 (0.66), 3.57 (0.17), and 0.41 (0.48), respectively. The Mann–Whitney *U* test showed a significant difference in urine KIM-1, IL-18, and IGFBP-7 levels between the two groups (Table 2).

**Table 2:** Biomarker values of urinary KIM-I, IL-18, and IGFBP-7 in septic patients with AKI and without AKI.

Biomarkers (ng/ml)	Sepsis		p*
	AKI	Without AKI	
KIM-I	* *	* *	0*.*000
Mean (SD)	1.92 (0.30)	0.96 (0.66)	
Median (min–max)	1.90 (1.25–2.55)	1.02 (0.00–2.00)	
IL-18			0*.*000
Mean (SD)	4.75 (0.71)	3.57 (0.17)	
Median (min–max)	4.79 (3.45–6.75)	3.59 (3.36–3.95)	
IGFBP-7			0*.*000
Mean	1.06 (0.59)	0.41 (0.48)	
Median (min–max)	1.05 (0.00–3.29)	0.14 (0.00–1.52)	

*Mann-Whitney Test AKI, acute kidney injury.

**Figure 3:**
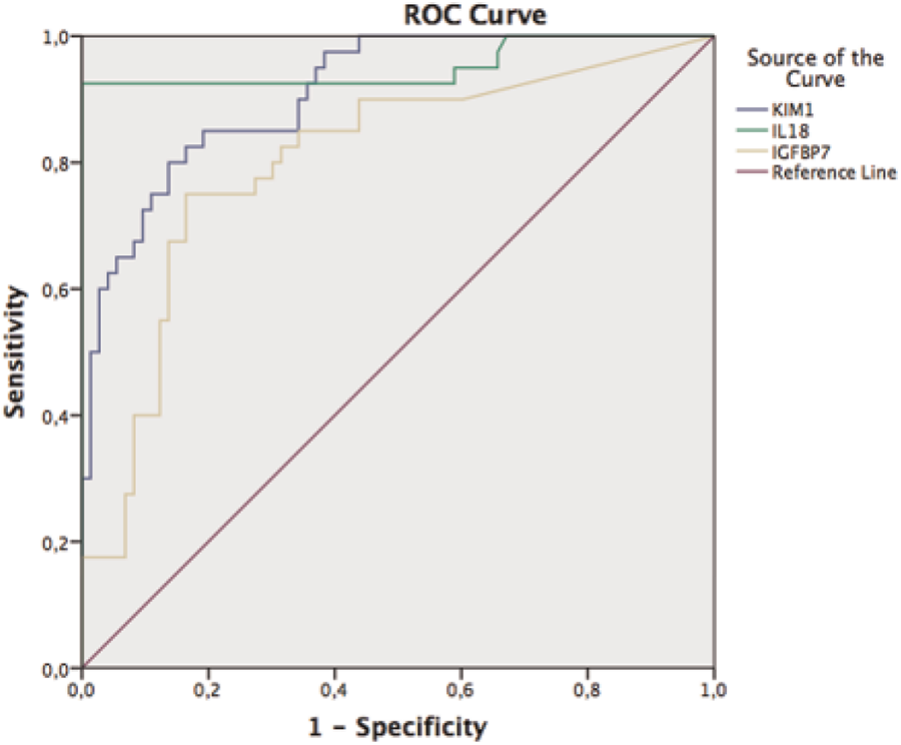
ROC curve urinary KIM-1, IL-18, and IGFBP-7.

ROC curve of KIM-1 urine with an AUC of 0.823 (well), *p* = 0.000, and 95% CI 0.738–0.909; urinary interleukin-18 with an AUC value of 0.921 (very good), *p* = 0.000, and 95% CI 0.862–0.981; and urinary IGFBP-7 with an AUC value of 0.752 (moderate), *p* = 0.000, and 95% CI 0.655–0.848 (Figure 3).

There was a significant relationship between urinary KIM-1 levels of 1.666 ng/ml and the occurrence of AKI in septic patients with *p*-value* *=* *0.000 and RR of 6.866 (95$ CI, 3.329–14.165), which means that septic patients with urinary KIM-1 levels ≥1.666 ng/ml have a higher risk of AKI than those with urine KIM-1 <1.666 ng/ml. There was also a significant relationship between urinary IL-18 levels ≥3.868 ng/ml with the occurrence of AKI in septic patients with *p *=* *0.000, RR 20.078 (95% CI, 6.593–61.142) which means that septic patients with urinary IL-18 levels ≥3.868 ng/ml have a higher risk of AKI compared to urinary IL-18 <3.868 ng/ml. Urinary IGFBP-7 levels ≥0.906 ng/ml and the occurrence of AKI in septic patients also showed a significant relationship where septic patients with IGFBP-7 levels ≥0.906 ng/ml have a higher risk of AKI than those with urine IGFBP-7 <0.906 ng/ml (Table 3).

**Table 3:** Cut-off point, relative risk, sensitivity, and specificity urinary KIM-I, IL-18, and IGFBP-7 in septic patients.

Biomarker cut-off point (ng/ml)	Sepsis		Sensitivity %	Specificity %	PPV %	NPV %	LR +	LR−	Accuracy (%)	P+	RR (95% CI)
	AKI (*n*)	Without AKI (*n*)									
KIM-1											
≥1*.*666	33	13	82*.*5	82*.*2	71*.*7	89*.*6	4*.*63	0*.*21	82*.*30	0*.*000	6.866 (3.329–14.165)
<1*.*666	7	60									
IL-18											
≥3*.*868	37	6	92*.*50	91*.*78	86*.*04	95*.*71	11*.*50	0*.*07	92*.*03	0*.*000	20.078 (6.593–61.142)
<3*.*865	3	67									
IGFBP-7											
≥0*.*906	30	18	75*.*00	75*.*34	62*.*50	84*.*61	3*.*12	0*.*33	75*.*22	0*.*000	4.063 (2.206–7.483)
<0*.*906	10	55	* *								

AKI, acute kidney injury; PPV, positive predictive value; NPV, negative predictive value; LR+, positive likelihood ratio; LR−, negative likelihood ratio; RR, relative risk; CI, confidence interval.

## Discussions

Early detection of AKI in septic patients is expected to reduce mortality and increase patient life expectancy. Sepsis is the most common cause of AKI. In children, sepsis caused 26%–50% of all AKI compared to 7%–10% of AKI caused by primary renal diseases (17). The incidence of AKI in septic patients admitted to the intensive care unit (ICU) ranges from 13% to 78%, depending on the severity of sepsis and the AKI criteria (18). AKI can be caused by a variety of factors, including sepsis, age, and primary disease (21). In this study, from 113 septic patients admitted to the PICU, AKI occurred in 40 patients (35.39%); this is related to the severity of sepsis, PELOD-2 score **≥**10, primary disease, and patients aged <5 years. Sepsis incidence was mostly found in children younger than 5 years (22). A previous study by Afroze reported septic patients with AKI were more common in ≤5-year-old patient than those older than 5 years old (23).

Urinary levels of KIM-1, IL-18, and IGFBP-7 were higher in septic patients with AKI than in septic patients without AKI. This study discovered that urinary KIM-1, IL-18, and IGFBP-7 levels increased first after ischemia-reperfusion injury, followed by an increase in plasma creatinine levels and a decrease in urine volume. Urine IL-18 biomarker (cut-off pint 3.868 ng/ml) detects the occurrence of AKI in septic patients on the first day of treatment in the PICU better than urine KIM-1 (cut-off point 1.666 ng/ml) and urinary IGFBP-7 (cut-off point 0.906 ng/ml). In septic patients, systemic inflammation causes the induction of proinflammatory cytokines (IL-1, IL-6, TNF-α), vasodilation of blood vessels, and microcirculation dysfunction, which leads to renal ischemia. Renal ischemia will cause microvascular injury, tubular injury, and inflammation (24).

Urinary IL-18 is an inflammatory mediator released by renal tubular cells after renal ischemic injury (25). During the disease phase, interactions between microvascular injury, tubular injury, and inflammation will cause urinary IL-18 excretion and GFR decrease. If this phase continues, it will cause AKI and increase the urinary IL-18 levels (26). A study on urinary IL-18 using different methods was also conducted by several previous researchers, including Ghorab et al., who reported that urinary IL-18 levels were higher in patients with AKI compared to patients without AKI. Urine IL-18 sensitivity was 91.1% and specificity was 93.9% 6 h after ICU admission. Urinary IL-18 levels are more specific and sensitive in predicting AKI than serum creatinine levels (26).

Urine IL-18 levels increase within 4–6 h, peak at 12 h, and remain significantly higher after 48 h in AKI patients. The specificity and sensitivity of urinary IL-18 are both higher than 90%. For early diagnosis of AKI, urinary IL-18 levels are strongly correlated with AKI risk, and its increase is closely related to the duration of AKI, implying that IL-18 can be used to monitor the progression of AKI (27, 28). Faisal reported that urinary IL-18 biomarkers at 6 h after sepsis had a sensitivity of 77.78%, specificity 82.60%, while at 48 h after sepsis had a sensitivity of 70.37% and specificity of 69.56% in detecting AKI (11).

According to a 2019 study by Khreba, KIM-1 is a well-known urinary biomarker for renal injury and has been evaluated as an early predictor of acute kidney injury in post-cardiopulmonary bypass in open heart surgery patients compared to routinely used serum creatinine (30).

IGFBP7 and TIMP-2 are proteins expressed in renal tubular cells during periods of cellular stress or injury, particularly in sepsis. A previous study mentioned that a [TIMP-2]* *x* *[IGFBP7] value >0.3 had a sensitivity of 92% for moderate or severe AKI in the next 12 h and was associated with approximately seven times the risk compared to values <0.3 in the Topaz study. Higher levels of [TIMP-2]* *x* *[IGFBP7] levels are more specific for assessing kidney injury; in a secondary analysis of the Topaz study, a cut-off of 2.0 was associated with specificity for moderate to severe AKI of 95%, although the sensitivity fell to 37% (31, 32).

Another study by Liu discovered that the diagnosis accuracy of IGFBP7 and TIMP-2 in diagnosing ARF in its early stage had a sensitivity of 83% (95% CI 0.75–0.89), specificity of 72% (95% CI 0.56–0.84), and summary receiver operating characteristic (SROC) of 0.86 (95% CI 0.82–0.88), while on stage 2 of GgGA according to the 2012 KDIGO classification, it had a sensitivity of 0.92 (95% CI 0.81–0.96), specificity of 0.63 (95% CI 0.49–0.74), and SROC of 0.88 (95% CI 0.85–0.91). Therefore, TIMP-2 and IGFBP7 are reliable for the early detection of GgGA (33).

A study by Lin et al. in 2012 showed that urine IL-18 levels in pediatric patients (<18 years) and early AKI predictive time (12 h) were more effective in predicting AKI, with diagnostic odds ratios of 7.51 (2.99–18.88) and 8.18 (2.19–30.51), respectively (34). It is important to determine the biomarker's cut-off point between the healthy children and the patient. According to the literature, the normal value of serum IL-18 in children and adults is 50–150 pg/ml (35). However, the normal value for urinary IL-18 in children was not found in the previous study**.**

## Conclusions

Urinary KIM-1, IL-8, and IGFBP-7 levels could be used to predict AKI in septic patients. Urinary IL-8 has a higher sensitivity and specificity as a predictor of AKI in patients with sepsis**.**

## Strengths and limitations

The strength of this study is that it used a prospective cohort study conducted in Wahidin Sudhirohusodo Hospital, which is the largest reference hospital in eastern Indonesia, so the patients with sepsis in this study were coming from most of areas in Eastern Indonesia. The patients treated in the PICU had the same severity and were treated and managed using the same protocol. Moreover, this is the first study in eastern Indonesia on biomarker levels of KIM-1, IL-18, and IGFBP-7 in septic patients with AKI**.**

The limitation of this study is that urinary KIM-1, IL18, and IGFBP-7 levels were not examined serially to determine the pattern of changes in their levels from time to time until AKI occurred. Serum creatinine levels are checked every two days, rather than every day. The urine test for all biomarkers was not performed in real time, but rather after several hours of storage. Another limitation is that there were no controls (non-septic patients), but we did include non-septic patients with AKI. Besides that, the primary diseases in this study are remarkably diverse. A multicenter study of urinary KIM-1, IL-18, and IGFBP-7 levels was recommended in septic patients admitted to the PICU to determine the dynamic pattern of changes in urinary biomarker levels. Moreover, similar research on other urinary biomarkers for predicting AKI in septic patients is still required. Urinary IL-18 biomarkers can be used to predict the occurrence of AKI in septic patients admitted to the PICU; therefore, urine IL-18 levels should be checked routinely in every septic patient admitted.

## Data Availability

The raw data supporting the conclusions of this article will be made available by the authors, without undue reservation.
